# The predictive value of participant subgroups in a temporary alcohol abstinence challenge: compliance with abstinence and changes in drinking behaviour

**DOI:** 10.1093/alcalc/agaf026

**Published:** 2025-05-22

**Authors:** Nathalie Kools, Andrea D Rozema, Dike van de Mheen, Rob H L M Bovens, Jolanda J P Mathijssen

**Affiliations:** Department of Tranzo Scientific Centre for Care and Wellbeing, Tilburg School of Social and Behavioral Sciences, Tilburg University, PO box 90153, 5000 LE Tilburg, the Netherlands; Department of Tranzo Scientific Centre for Care and Wellbeing, Tilburg School of Social and Behavioral Sciences, Tilburg University, PO box 90153, 5000 LE Tilburg, the Netherlands; Department of Tranzo Scientific Centre for Care and Wellbeing, Tilburg School of Social and Behavioral Sciences, Tilburg University, PO box 90153, 5000 LE Tilburg, the Netherlands; Department of Tranzo Scientific Centre for Care and Wellbeing, Tilburg School of Social and Behavioral Sciences, Tilburg University, PO box 90153, 5000 LE Tilburg, the Netherlands; Positive Lifestyle Foundation, Ringbaan-Oost 298, 5018 AL Tilburg, the Netherlands; Department of Tranzo Scientific Centre for Care and Wellbeing, Tilburg School of Social and Behavioral Sciences, Tilburg University, PO box 90153, 5000 LE Tilburg, the Netherlands

**Keywords:** alcohol, temporary alcohol abstinence, latent class analysis, alcohol drinking

## Abstract

**Introduction:**

Dividing participants of Temporary alcohol Abstinence Challenges (TACs) into subgroups can improve intervention effectiveness by identifying individuals who require extra support. In a previous study, participant subgroups were identified based on determinants of behaviour change, including drinking refusal self-efficacy, craving, and behavioural automaticity. However, the predictive value of these subgroups for TAC success remains unknown. This study examined their predictive value for (i) abstinence during a TAC and (ii) changes in drinking behaviour.

**Methods:**

Data were analysed from 1800 Dutch TAC participants who completed baseline and eight-month follow-up questionnaires. Binary logistic regression assessed the effect of subgroup on abstinence. Ordinal and binary logistic regressions within Generalized Estimating Equation models examined subgroup effects on drinking behaviour changes, including drinking frequency, glasses per drinking day, and excessive volumes.

**Results:**

TAC subgroups differed in abstinence and in changes in drinking frequency and glasses per drinking day on weekdays. ‘Habitual drinkers with perceived control to refuse’ and ‘drinkers in control’ were more likely to abstain during the challenge than ‘ordinary drinkers’, whereas ‘drinkers not in control’ were less likely to abstain. ‘Drinkers in control’ showed smaller reductions in drinking frequency and glasses per drinking day on weekdays compared with ‘ordinary drinkers’. No significant differences were found in changes in excessive drinking volumes and glasses per drinking day on weekends.

**Conclusions:**

This study demonstrated the predictive value of subgroups for abstinence but found limited predictive value for changes in drinking behaviour after the challenge. Future research could explore personalized support to optimize behaviour change.

## Teaser

**Short Summary:** The study examined subgroups of Temporary alcohol Abstinence Challenge (TAC) participants. While subgroups predicted abstinence during the TAC, they had limited value in predicting postchallenge changes in drinking behaviour. The participation value for two subgroups may be questionable. Future research could explore personalized support to optimize behaviour change.

## Introduction

Alcohol use is a major global risk factor contributing to disease burden and causing physical, mental, and social harms (Rehm 2011, Castillo-Carniglia et al. 2019, Murray et al. 2020). It represents a significant public health challenge. Reducing per capita alcohol use is strongly linked to decreased alcohol-related harm across the entire drinking population (Rossow and Mäkelä 2021). However, although reducing alcohol use is beneficial, many individuals struggle to do so (Bartram et al. 2017, Charlet and Heinz 2017). One promising strategy for preventing alcohol-related harm may be Temporary alcohol Abstinence Challenges (TACs).

TAC participants voluntarily abstain from alcohol for a specified period, typically 1 month. In recent years, TACs have gained popularity, with various organized challenges available for participants to join. Their aims range from fundraising (e.g. Dry July and DryFeb) to awareness and behaviour change focussed (e.g. IkPas and Dry January) (de Ternay et al. 2022, Butters et al. 2023), with some TACs offering additional support, such as newsletters, personal dashboards, and forums (Bovens et al. 2020, de Visser and Nicholls 2020).

One-month TACs have been associated with reduced alcohol use after 6 months, highlighting their potential public health impact (de Visser et al. 2016, Thienpondt et al. 2017, de Visser and Piper 2020). The exact mechanisms of long-term behaviour change remain unclear. However, various factors have been proposed, including increased self-efficacy, changes in habits, shifts in social norms, alternations in alcohol-related identity, experienced benefits of abstinence (e.g. money savings, weight loss, and sleep improvement), and greater awareness of drinking patterns after participation (de Visser and Nicholls 2020, Butters et al. 2023, Thienpondt et al. 2025). Indeed, prospective studies showed improved drinking-refusal self-efficacy (i.e. perceived ability to refuse alcohol), wellbeing, and mental and physical health at 1-month follow-ups (de Visser and Nicholls 2020, de Visser and Piper 2020). Longer-term reductions in alcohol use were observed in all TAC participants, with larger reductions among those who successfully stayed abstinent during the challenge (i.e. generally 61%–64% of participants). Abstinence during TACs was predicted by male gender, lower alcohol use, higher drinking refusal self-efficacy, and greater use of email support (de Visser et al. 2016, de Visser and Nicholls 2020, de Visser and Piper 2020). Moreover, the psychological and behavioural changes observed among TAC participants were not found in comparator samples of the general population (de Visser and Piper 2020, Rossow and Mäkelä 2021).

A previous study identified subgroups of TAC participants to gain deeper insights into participant characteristics and help improve intervention effectiveness by better targeting individuals and identifying those requiring additional support (Kools et al. 2024). Using latent class analysis (LCA), four subgroups of participants were identified based on various potential determinants of changes in drinking behaviour, including drinking refusal self-efficacy (i.e. with the domains social pressure, emotional regulation, and opportunistic drinking), craving (i.e. strong desire to drink), and behavioural automaticity (i.e. habit-driven drinking with little conscious effort).

Each subgroup exhibited distinct configurations of determinants, assessed on a 5-point scale. Subgroup 1 was labelled ‘ordinary drinkers’ because it comprised approximately half of the study sample (49.0%) and had mean scores that were closest to the full study sample average across the determinants. Mean scores on drinking refusal self-efficacy were significantly lower than those in Subgroup 2 and Subgroup 3 but higher than Subgroup 4. Craving and behavioural automaticity scores in Subgroup 1 were slightly above full sample average and significantly lower than Subgroup 4 but higher than Subgroup 2. Subgroup 2 (21.4%) was labelled ‘drinkers in control’ due to the relative ‘control’ this subgroup showed in comparison to other subgroups: they, along with Subgroup 3, had significantly higher drinking refusal self-efficacy scores than Subgroups 1 and 4, and their craving and behavioural automaticity scores were the lowest among all subgroups. Subgroup 3 (18.4%) was labelled ‘habitual drinkers with perceived control to refuse’ because, like Subgroup 2, they had higher self-efficacy scores than Subgroups 1 and 4. However, their craving and automaticity scores were near the full sample average, higher than Subgroup 2 and lower than Subgroup 4. Descriptive characteristics showed that this subgroup had relatively higher alcohol use (e.g. 86.1% drank four or more days per week, and 54.3% drank excessive volumes) and higher previous participation in organized TACs (86.1% compared with 66.3%–69.6% in other classes). Finally, Subgroup 4 (11.2%), labelled ‘drinkers not in control’, exhibited the most extreme mean scores, suggesting a relative lack of control compared with the other subgroups. This subgroup had significantly lower scores on drinking refusal self-efficacy and higher scores on craving and behavioural automaticity compared with the other classes (Kools et al. 2024).

For the first time, research has been conducted into subgroups of TAC participants, addressing a previously unexplored area (Kools et al. 2024). Therefore, there is a lack of knowledge regarding the predictive value of these subgroups (i.e. their distinctive subgroup characteristics) for TAC success, such as differential effects on abstinence or changes in drinking behaviour after the challenge. This knowledge could improve intervention effectiveness by identifying individuals needing extra support, as tailored interventions are more effective than one-size-fits-all approaches (Norman et al. 2007, Neville et al. 2009, Boumans et al. 2022).

This study aimed to examine the effect of previously identified participant subgroups on (i) abstinence during a TAC and (ii) changes in drinking frequency, the number of glasses used per drinking day (on weekdays and weekends), and excessive drinking volume after the challenge.

## Methods

### Study design and participants

Data were used from a longitudinal questionnaire study of the Dutch TAC ‘NoThanks!’ (Dutch: IkPas). Quantitative data were used from participants of the January TAC, also known as Dry January, which consisted of a baseline measurement (prechallenge, T0) and an 8-month follow-up (postchallenge, T1). T0 data were collected between 27 and 31 December 2021 and T1 data between 2 and 26 September 2022. Thirteen thousand seven hundred fifty-three participants registered before 1 January 2022, of whom 3803 completed the baseline measurement. Out of those who completed T0, 1815 participants completed the 8-month follow-up questionnaire (attrition rate = 47.7%). For the analyses, only participants were included who completed both questionnaires. Furthermore, due to our control variables, we excluded participants that scored ‘other, or do not want to say’ or had missing data on the gender variable (*n* = 5 and *n* = 2, respectively). Also, we excluded those with missing data on the abstinence variable (*n* = 8). Therefore, 1800 participants were used for the analyses.

The study sample (*n* = 1800) differed from those who only completed the baseline questionnaire (*n* = 1988). This sample was more likely to be older, retired, highly educated, and drink 0.5–2 glasses per drinking day and nonexcessive volumes. Additionally, they were less likely to be employed, had secondary education less often, and were less likely to drink five or more glasses per drinking day on both weekdays and weekends. Furthermore, they were more likely to be ‘habitual drinkers with perceived control to refuse’ and less likely to be ‘ordinary drinkers’.

### Procedure

Adults (≥18 years) were able to register online (IkPas.nl) to join the January TAC (i.e. ‘Dry January’), through which additional support could be accessed, such as motivational newsletters, personal dashboards, and forums. All registrants received an email with an invitation to complete the online questionnaire. Respondents provided informed consent, allowing their data to be used for research purposes and agreeing to be contacted for follow-up questionnaires. Ethical approval for the study was granted by the Ethics Review Board of Tilburg University (EC-2018.01).

### Measures

#### Latent class analysis subgroups

Subgroups comprised the four subgroups of TAC participants that were identified in a previous LCA of this research group: Subgroups 1 (‘ordinary drinkers’), 2 (‘drinkers in control’), 3 (‘habitual drinkers with perceived control to refuse’), and 4 ‘drinkers not in control’) (Kools et al. 2024). More detailed subgroup descriptions can be found in Kools et al. (2024).

#### Outcome variables

Abstinence during the TAC was measured with a self-developed item that asked participants to self-report on how many days of the challenge they drank alcohol, on a scale from 1 (0 days) to 6 (6 days or more). For the present study, this variable was dichotomized into ‘nonabstinence’ (scores of 1 to 6 drinking days or more) and ‘abstinence’ (score of 0 drinking days).

Drinking behaviour was assessed by drinking frequency, number of glasses per drinking day on weekdays (Monday–Thursday) and weekends (Friday–Sunday), and excessive drinking volumes. For drinking frequency, participants reported how many days they drank during the week, choosing from: ‘I never drink during weekdays/weekends’, ‘less than 1 day’, ‘1 day’, ‘2 days’, ‘3 days’, and ‘4 days’ (presented for the weekday question only). For glasses per drinking day and excessive drinking volumes, respondents indicated the number of glasses consumed on drinking days over the past 6 months, ranging from 0 to 6, and response options ‘7–9 glasses’ and ‘≥10 glasses’ for higher amounts. A standard glass in the Netherlands contains 10 g of alcohol (Central Bureau of Statistics (CBS) 2021).

For the present study, drinking frequency was determined by summing the number of days on which alcohol was used during weekdays and weekends, categorized as ‘never’, ‘1 day per week’, ‘2–3 days per week’, and ‘≥4 days per week’. Glasses per drinking day were categorized as ‘0 glasses’, ‘0.5–2 glasses’, ‘3–4 glasses’, ‘5–6 glasses’, and ‘≥7 glasses’ for both weekdays and weekends. As the dataset was predefined by the campaign organizers and based on ordinal scales, we were unable to combine weekday and weekend drinking patterns and therefore this distinction between in our measurements. Excessive drinking volume was determined by copying answers from 0 to 6 glasses, and recoding ‘7–9 glasses’ and ‘≥10 glasses’ as 7 and 10 glasses, respectively. Then, the average numbers of glasses per weekday and weekends were calculated by multiplying the number of drinking days with the average number of glasses (both calculated for weekdays and weekends). These two numbers were summed to obtain the average number of glasses per week and were categorized as ‘nonexcessive’ (≤14 glasses for women, others, or missing data; ≤21 glasses for men) or ‘excessive’ (>14 glasses for women, others, or missing data; >21 glasses for men), following Dutch national alcohol survey definitions (Central Bureau of Statistics (CBS) 2021).

#### Sociodemographic characteristics

Sociodemographic characteristics included age, gender, educational level, and employment status. Gender was categorized as ‘man’, ‘woman’, and ‘other, or prefer not to answer’. Educational level was categorized as ‘low’ (primary school, intermediate secondary), ‘secondary’ (higher secondary education/preparatory university education), and ‘high’ (higher education). Employment status was categorized as ‘employed’, ‘retired’, ‘student’, and ‘unemployed’.

### Analyses

All analyses were conducted using Statistical Product and Service Solutions (SPSS) software 27.0 (George and Mallery 2019). First, the estimated latent class model parameters of the subgroup classification, as determined in Kools et al. (2024), were used to classify the pre-challenge data (T0) in SPSS. In Latent GOLD 6.0, classification probabilities were reformulated as a set of logistic equations (Vermunt and Magidson 2021). These equations were applied to the data in SPSS, where each individual was assigned to a latent class based on the highest posterior membership probability (i.e. modal assignment). Second, logistic regressions were conducted. The first research aim was tested using binary logistic regression, with abstinence as the dependent variable, subgroup as the predictor, and gender and excessive drinking volume at T0 as control variables. Given previous research suggesting that abstinence during a TAC is associated with factors such as gender and baseline alcohol use (de Visser et al. 2016, de Visser and Nicholls 2020, de Visser and Piper 2020), additional analyses tested interaction effects between subgroup and gender and between subgroup and excessive drinking volume at T0.

To test the second research aim, ordinal logistic regressions were conducted on the ordinal variables ‘drinking frequency’, ‘glasses per drinking day on weekdays’, and ‘glasses per drinking day on weekends’ using the Generalized Estimating Equation (GEE) model. A binary logistic regression was conducted on the binary variable ‘excessive drinking volume’ using GEE. Analyses for all outcome variables included subgroup, the within-factor of time, its interaction, and two control variables (i.e. gender and abstinence). Building on prior research suggesting that gender and abstinence influence long-term alcohol use reductions in TACs (de Visser et al. 2016, de Visser and Nicholls 2020, de Visser and Piper 2020), additional analyses tested two extra interaction effects: (i) between subgroup, time, and gender and (2) between subgroup, time, and abstinence. Drinking frequency and the number of glasses per drinking day were analysed separately rather than relying solely on the binary variable ‘excessive drinking volume’, as changes in these measures provide a more sensitive and detailed indication of shifts in alcohol use patterns, which are strongly associated with public health impact and alcohol-related harm reduction (Rossow and Mäkelä 2021). For all analyses, no corrections for multiple comparisons were applied to avoid increasing the risk of false negatives, as the primary aim was to explore differences between subgroups rather than all group comparisons.

## Results

### Participants’ characteristics

The average age of participants was 56.63 years (*SD* = 11.11). The majority were female (63.4%), highly educated (73.7%), employed (69.4%), and categorized in the subgroup ‘ordinary drinkers’ (45.0%). Regarding drinking behaviour at T0, the majority had a higher drinking frequency (e.g. 68.7% drank four or more days per week), drank 0.5–2 glasses per drinking day on weekdays (44.4%), and 3–4 glasses per drinking day on weekends (43.2%). A total of 71.2% of participants remained abstinent during the TAC. Other participants’ characteristics are shown in Table 1.

**Table 1:** Participant characteristics (*n* = 1800).

**Variables**		*n (%)*	
Age *M* (in years) (*SD*)		56.63 (11.11)	
Gender	Female	1142 (63.4)	
	Male	658 (36.6)	
Educational level	Low	114 (6.4)	
	Secondary	357 (19.9)	
	High	1323 (73.7)	
Employment status	Employed	1245 (69.4)	
	Retired	437 (24.4)	
	Student	29 (1.6)	
	Unemployed	83 (4.6)	
Subgroup	1 ‘ordinary drinkers’	810 (45.0)	
	2 ‘drinkers in control’	339 (18.8)	
	3 ‘habitual drinkers with perceived control to refuse’	488 (27.1)	
	4 ‘drinkers not in control’	163 (9.1)	
Abstinence during challenge		1281 (71.2)	
		**At baseline (T0)**	**At follow-up (T1)**
Drinking frequency	Never	34 (1.9)	10 (.6)
	<2 days per week	116 (6.4)	193 (11.4)
	2 to 3 days per week	413 (22.9)	547 (32.4)
	≥4 days per week	1237 (68.7)	940 (55.6)
Glasses per drinking day (weekdays)	0 glasses	183 (10.2)	220 (13.0)
	0.5–2 glasses	800 (44.4)	794 (47.0)
	3–4 glasses	567 (31.5)	507 (30.0)
	5–6 glasses	165 (9.2)	113 (6.7)
	≥ 7 glasses	85 (4.7)	56 (3.3)
Glasses per drinking day (weekends)	0 glasses	36 (2.0)	220 (13.0)
	0.5–2 glasses	457 (25.4)	794 (47.0)
	3–4 glasses	777 (43.2)	507 (30.0)
	5–6 glasses	336 (18.7)	113 (6.7)
	≥ 7 glasses	194 (10.8)	56 (3.3)
Excessive drinking volume^a^		713 (39.6)	480 (28.4)

*Note.* M, mean; SE, standard error.

^a^Excessive drinking volume = >14 (women, other or missing) or >21 (men) glasses per week.

Note that there was an attrition rate of 47.7% from T0, and various variables had multiple missing data (ranging from 2 to 12 data points and drinking behaviour variables having 110 missing data points on T1).

### Abstinence

Table 2 presents the results of the analyses on abstinence during the challenge. TAC subgroups differed in their abstinence rates, with 65.0% of ‘ordinary drinkers’, 74.8% of ‘drinkers in control’, 8.3% of ‘habitual drinkers with perceived control to refuse’, and 52.0% of ‘drinkers not in control’ remaining abstinent. More specifically, ‘drinkers in control’ and ‘habitual drinkers with perceived control to refuse’ were more likely to remain abstinent than ‘ordinary drinkers’. ‘Drinkers not in control’ were less likely to remain abstinent than ‘ordinary drinkers’. Furthermore, participants who drank nonexcessive volumes at T0 were more likely to remain abstinent than those who drank excessive volumes.

**Table 2:** Results of binary logistic regression models on abstinence during the challenge (*n* = 1800).

**Variables**	**Odds ratio (95% Confidence Interval)**
Subgroup (ref: subgroup 1)	
Subgroup 2	1.52 (1.12–2.08)^*^^*^
Subgroup 3	2.12 (1.62–2.77)^*^^*^^*^
Subgroup 4	0.67 (0.47–0.95)^*^
Gender (ref: female)	
Male	1.24 (1.00–1.55)
Excessive drinking volume (ref: excessive)	
Nonexcessive^a^	1.47 (1.17–1.84)^*^^*^^*^

*Note.* Ref, reference group. Subgroup 1 = ‘ordinary drinkers’. Subgroup 2 = ‘drinkers in control’. Subgroup 3 = ‘habitual drinkers with perceived control to refuse’. Subgroup 4 = ‘drinkers not in control’.

^*^ *P <* .05.

^**^ *P* < .01.

^***^ *P* < .001.

^a^Excessive drinking volume = >14 (women, other or missing) or >21 (men) glasses per week.

Additional analyses showed no significant interaction effects: the association between subgroup and abstinence did not depend on gender [Wald *χ*^2^(3) = 2.66, *P* = .446] or drinking volume [Wald *χ*^2^(3) = 1.22, *P* = .749].

### Changes in drinking behaviour

#### Drinking frequency

Table 3 presents the frequencies and proportions of drinking frequency per subgroup at each time point (T0 and T1), and Table 4 presents the results of the analyses on changes in drinking frequency from prechallenge to 8-month follow-up. There were significant effects of subgroup, time, gender, and abstinence on drinking frequency. More specifically, ‘drinkers in control’ and ‘habitual drinkers with perceived control to refuse’ scored lower on drinking frequency compared with ‘ordinary drinkers’. ‘Drinkers not in control’ scored higher on drinking frequency compared with ‘ordinary drinkers’. Postchallenge drinking frequency was significantly lower than prechallenge. Males scored higher on drinking frequency compared with females, and nonabstinent participants scored higher on drinking frequency compared with abstinent participants.

**Table 3:** Drinking frequency proportions per subgroup at each time point (T0 and T1).

**Subgroup**	**Drinking frequency**	**Baseline (T0) (*n* = 1800)** *n* (%)	**Follow-up (T1) (*n* = 1690** ^**a**^ **)** *n* (%)
Subgroup 1 (‘ordinary drinkers’)	Never	0 (.0)	5 (.6)
	Less than 2 days per week	27 (3.3)	63 (8.1)
	2 or 3 days per week	152 (18.8)	228 (29.3)
	4 or more days per week	631 (77.9)	481 (61.9)
	*Total*	*810 (100.0)*	*777 (100.0)*
Subgroup 2 (‘drinkers in control’)	Never	26 (7.7)	2 (.7)
	Less than 2 days per week	67 (19.8)	84 (27.8)
	2 or 3 days per week	142 (41.9)	143 (47.4)
	4 or more days per week	104 (30.7)	73 (24.2)
	*Total*	*339 (100.0)*	*302 (100.0)*
Subgroup 3 (‘habitual drinkers with perceived control to refuse’)	Never	6 (1.2)	3 (.7)
	Less than 2 days per week	21 (4.3)	41 (8.9)
	2 or 3 days per week	107 (21.9)	152 (33.0)
	4 or more days per week	354 (72.5)	265 (57.5)
	*Total*	*488 (100.0)*	*461 (100.0)*
Subgroup 4 (‘drinkers not in control’)	Never	2 (1.2)	0 (.0)
	Less than 2 days per week	1 (.6)	5 (3.3)
	2 or 3 days per week	12 (7.4)	24 (16.0)
	4 or more days per week	148 (90.8)	121 (80.7)
	*Total*	*163 (100.0)*	*150 (100.0)*

*Note.* The italicized values in the ``Total'' rows indicate the number of participants in each subgroup at either the baseline or follow-up measurement. ^a^Drinking frequency data were missing for 110 respondents on T1.

**Table 4:** Ordinal logistic regression by generalized estimating equations for longitudinal drinking frequency data (*n* = 1800)*.*

**Variables**	**Odds ratio (95% confidence interval)**
Outcome (ref: 4 or more days per week)	
Never	0.01 (.00–0.01)^*^^*^^*^
Less than 2 days per week	0.05 (.04–0.06)^*^^*^^*^
2 or 3 days per week	0.32 (.27–0.39)^*^^*^^*^
Subgroup (ref: subgroup 1)	
Subgroup 2	0.11 (.09–0.15)^*^^*^^*^
Subgroup 3	0.76 (.58–0.98)^*^
Subgroup 4	2.67 (1.53–4.66)^*^^*^^*^
Time (ref: prechallenge)	
Postchallenge	0.45 (.38–0.53)^*^^*^^*^
Subgroup*Time (ref: subgroup 1 and postchallenge)	
Subgroup 2*postchallenge	1.95 (1.52–2.52)^*^^*^^*^
Subgroup 3*postchallenge	1.14 (.88–1.49)
Subgroup 4*postchallenge	0.94 (.54–1.64)
Gender (female as reference)	
Male	1.20 (1.01–1.44)^*^
Abstinence (ref: abstinence)	
Nonabstinence	1.28 (1.05–1.55)^*^

*Note*. Instead of the intercept, SPSS uses the categories of the data for the ordinal regression. Ref, reference group. Subgroup 1 = ‘ordinary drinkers’. Subgroup 2 = ‘drinkers in control’. Subgroup 3 = ‘habitual drinkers with perceived control to refuse’. Subgroup 4 = ‘drinkers not in control’.

^*^ *P* < .05.

^**^ *P* < .01.

^***^ *P* < .001.

Furthermore, there was a significant interaction effect, showing that the change in drinking frequency over time differed between the four subgroups. More specifically, ‘drinkers in control’ showed a significantly smaller reduction in drinking frequency compared with ‘ordinary drinkers’. Additional analyses showed no significant interaction effects between subgroup, time, and gender [Wald *χ*^2^(7) = 3.34, *P* = .852], nor between subgroup, time, and abstinence [Wald *χ*^2^(7) = 1.40, *P* = .167].

### Glasses per drinking day

Table 5 presents the frequencies and proportions of glasses per drinking day (on weekdays and weekends) per subgroup at each time point (T0 and T1), and Table 6 presents the results of the analyses on changes in glasses per drinking day from prechallenge to 8-month follow-up.

**Table 5:** Glasses per drinking day (on weekdays and weekends) proportions per subgroup at each time point (T0 and T1).

**Subgroup**	**Glasses per drinking day**	**Weekdays** ^**a**^		**Weekends** ^**b**^	
		**Baseline (T0) (*n* = 1800)** *n* (%)	**Follow-up (T1)** **(*n* = 1690)** *n* (%)	**Baseline (T0) (*n* = 1800)** *n* (%)	**Follow-up (T1)** **(*n* = 1690)** *n* (%)
Subgroup 1	0 glasses	39 (4.8)	77 (9.9)	1 (.1)	77 (9.9)
	0.5–2 glasses	341 (42.1)	343 (44.1)	161 (19.9)	343 (44.1)
	3–4 glasses	303 (37.4)	270 (34.7)	377 (46.5)	270 (34.7)
	5–6 glasses	86 (10.6)	59 (7.6)	174 (21.5)	59 (7.6)
	≥7 glasses	41 (5.1)	28 (3.6)	97 (12.0)	28 (3.6)
	*Total*	*810 (100.0)*	*777 (100.0)*	*810 (100.0)*	*777 (100.0)*
Subgroup 2	0 glasses	100 (29.5)	77 (25.5)	27 (8.0)	77 (25.5)
	0.5–2 glasses	192 (56.6)	184 (60.9)	170 (50.1)	184 (60.9)
	3–4 glasses	37 (10.9)	35 (11.6)	101 (29.8)	35 (11.6)
	5–6 glasses	6 (1.8)	4 (1.3)	25 (7.4)	4 (1.3)
	≥7 glasses	4 (1.2)	2 (.7)	16 (4.7)	2 (.7)
	*Total*	*339 (100.0)*	*302 (100.0)*	*339 (100.0)*	*302 (100.0)*
Subgroup 3	0 glasses	37 (7.6)	57 (12.4)	6 (1.2)	57 (12.4)
	0.5–2 glasses	225 (46.1)	225 (48.8)	111 (22.7)	225 (48.8)
	3–4 glasses	164 (33.6)	139 (30.2)	242 (49.6)	139 (30.2)
	5–6 glasses	48 (9.8)	30 (6.5)	88 (18.0)	30 (6.5)
	≥7 glasses	14 (2.9)	10 (2.2)	41 (8.4)	10 (2.2)
	*Total*	*448 (100.0)*	*461 (100.0)*	*488 (100.0)*	*461 (100.0)*
Subgroup 4	0 glasses	7 (4.3)	9 (6.0)	2 (1.2)	9 (6.0)
	0.5–2 glasses	42 (25.8)	42 (28.0)	15 (9.2)	42 (28.0)
	3–4 glasses	63 (38.7)	63 (42.0)	57 (35.0)	63 (42.0)
	5–6 glasses	25 (15.3)	20 (13.3)	49 (30.1)	20 (13.3)
	≥7 glasses	26 (16.0)	16 (10.7)	40 (24.5)	16 (10.7)
	*Total*	*163 (100.0)*	*150 (100.0)*	*163 (100.0)*	*150 (100.0)*

*Note.* Subgroup 1 = ‘ordinary drinkers’. Subgroup 2 = ‘drinkers in control’. Subgroup 3 = ‘habitual drinkers with perceived control to refuse’. Subgroup 4 = ‘drinkers not in control’. The italicized values in the ``Total'' rows indicate the number of participants in each subgroup at either the baseline or follow-up measurement.

^a^Weekdays = Monday–Thursday.

^b^Weekends = Friday–Sunday.

**Table 6:** Ordinal logistic regression by generalized estimating equations for longitudinal data on glasses per drinking day (on weekdays and weekends) (*n* = 1800).

**Variables**	**Odds ratio (95% confidence interval)**	
	**Weekdays** ^a^	**Weekends** ^b^
Outcome (ref: ≥7 glasses per drinking day)		
0 glasses per drinking day	0.09 (.07–0.10)^*^^*^^*^	.02 (.02–0.03)^*^^*^^*^
0.5–2 glasses per drinking day	1.17 (1.00–1.37)^*^	0.35 (.30–0.42)^*^^*^^*^
3–4 glasses per drinking day	7.47 (6.25–8.92)^*^^*^^*^	2.69 (2.30–3.15)^*^^*^^*^
5–6 glasses per drinking day	25.45 (20.21–32.03)^*^^*^^*^	9.93 (8.26–11.95)^*^^*^^*^
Subgroup (ref: subgroup 1)		
Subgroup 2	0.15 (.12–0.20)^*^^*^^*^	.21 (.16–0.27)^*^^*^^*^
Subgroup 3	0.77 (.63–0.95)^*^	0.77 (.63–0.94)^*^
Subgroup 4	2.20 (1.58–3.05)^*^^*^^*^	2.20 (1.63–2.98)^*^^*^^*^
Time (prechallenge as reference)		
Postchallenge	0.69 (.61–0.77)^*^^*^^*^	.21 (.18–0.24)^*^^*^^*^
Subgroup*Time (ref: subgroup 1 and postchallenge)		
Subgroup 2*postchallenge	1.62 (1.28–2.06)^*^^*^^*^	1.12 (.83–1.52)
Subgroup 3*postchallenge	1.00 (.82–1.23)	0.98 (.78–1.24)
Subgroup 4*postchallenge	1.05 (.79–1.40)	1.12 (.80–1.57)
Gender (female as reference)		
Male	1.69 (1.43–2.00)^*^^*^^*^	1.90 (1.62–2.22)^*^^*^^*^
Abstinence (ref: abstinence)		
Nonabstinence	1.48 (1.24–1.76)^*^^*^^*^	1.37 (1.17–1.62)^*^^*^^*^

*Note*. Instead of the intercept, SPSS uses the categories of the data for the ordinal regression. Ref, reference group. Subgroup 1 = ‘ordinary drinkers’. Subgroup 2 = ‘drinkers in control’. Subgroup 3 = ‘habitual drinkers with perceived control to refuse’. Subgroup 4 = ‘drinkers not in control’.

^*^ *P* < .05.

^**^ *P* < .01.

^***^ *P* < .001.

^a^Weekdays = Monday–Thursday.

^b^Weekends = Friday–Sunday.

There were significant effects of subgroup, time, gender, and abstinence on glasses per drinking day, with similar effect directions on both weekdays and weekends. More specifically, ‘drinkers in control’ and ‘habitual drinkers with perceived control to refuse’ scored lower on glasses per drinking day compared with ‘ordinary drinkers’. ‘Drinkers not in control’ scored higher on glasses per drinking day. Postchallenge glasses per drinking day were significantly lower than prechallenge. Additionally, males scored higher on glasses per drinking day compared with females, and nonabstinent participants scored higher compared with abstinent participants.

Moreover, there was a significant interaction effect between subgroup and time on glasses per drinking day on weekdays. The interaction effect for weekends was not significant, [*χ*^2^(3) = 1.11, *P* = .775]. Thus, subgroups only significantly differed in changes in glasses per drinking day on weekdays, with ‘drinkers in control’ having a significantly smaller reduction in glasses per drinking day compared with ‘ordinary drinkers’. Additional analyses showed no significant interaction effects on glasses per drinking day (on both weekdays and weekends) between subgroup, time, and gender, [Wald *χ*^2^(7) = 7.73, *P* = .357, and Wald *χ*^2^(7) = 13.71, *P* = .057, respectively], nor between subgroup, time, and abstinence [Wald *χ*^2^(7) = 8.96, *P* = .256 and Wald *χ*^2^(7) = 8.94, *P* = .257, respectively].

### Excessive drinking volume

Table 7 presents the frequencies and proportions of excessive drinking volume per subgroup at each time point (T0 and T1), and Table 8 presents the results of the analyses on changes in excessive drinking volume from prechallenge to 8-month follow-up. There was a significant effect of subgroup, time, gender, and abstinence. ‘Drinkers in control’ and ‘habitual drinkers with perceived control to refuse’ were less likely to drink excessive volumes compared with ‘ordinary drinkers’. ‘Drinkers not in control’ were more likely to drink excessive volumes compared with ‘ordinary drinkers’. Postchallenge excessive drinking volume proportions were significantly lower than prechallenge proportions. Additionally, males were less likely to drink excessive volumes (i.e. note that excessive volume labels were gender dependent) compared with females, and nonabstinent participants were more likely to drink excessive volumes compared with abstinent participants.

**Table 7:** Excessive drinking volume proportions per subgroup at each time point (T0 and T1).

**Subgroup**	**Drinking volume**	**Baseline (T0) (*n* = 1800)** *n* (%)	**Follow-up (T1) (*n* = 1690)** *n* (%)
Subgroup 1	Nonexcessive	423 (52.2)	521 (67.1)
	Excessive	387 (47.8)	256 (32.9)
	*Total*	*810 (100.0)*	*777 (100.0)*
Subgroup 2	Nonexcessive	317 (93.5)	288 (95.4)
	Excessive	22 (6.5)	14 (4.6)
	*Total*	*339 (100.0)*	*302 (100.0)*
Subgroup 3	Nonexcessive	298 (61.1)	339 (73.5)
	Excessive	190 (38.9)	122 (26.5)
	*Total*	*488 (100.0)*	*461 (100.0)*
Subgroup 4	Nonexcessive	49 (30.1)	62 (41.3)
	Excessive	114 (69.9)	88 (58.7)
	*Total*	*163 (100.0)*	*150 (100.0)*

*Note.* Subgroup 1 = ‘ordinary drinkers’. Subgroup 2 = ‘drinkers in control’. Subgroup 3 = ‘habitual drinkers with perceived control to refuse’. Subgroup 4 = ‘drinkers not in control’. The italicized values in the ``Total'' rows indicate the number of participants in each subgroup at either the baseline or follow-up measurement. Excessive drinking volume = >14 (women, other, or missing) or >21 (men) glasses per week.

**Table 8:** Binary regression by generalized estimating equations for longitudinal data on excessive drinking volumes (*n* = 1800).

**Variables**	**Odds ratio (95% confidence interval)**
Subgroup (ref: subgroup 1)	
Subgroup 2	0.08 (.05–0.12)^*^^*^^*^
Subgroup 3	0.74 (.58–0.93)^*^
Subgroup 4	2.57 (1.77–3.72)^*^^*^^*^
Time (ref: prechallenge)	
Post-challenge	0.53 (0.46–0.60)^*^^*^^*^
Subgroup*Time (ref: subgroup 1 and postchallenge)	
Subgroup 2*postchallenge	1.32 (.73–2.39)
Subgroup 3*postchallenge	1.05 (.83–1.33)
Subgroup 4*postchallenge	1.14 (.82–1.60)
Gender (female as reference)	
Male	0.55 (0.45–0.67)^*^^*^^*^
Abstinence (ref: abstinence)	
Nonabstinence	1.28 (1.04–1.57)^*^

*Note*. Ref, reference group. Subgroup 1 = ‘ordinary drinkers’. Subgroup 2 = ‘drinkers in control’. Subgroup 3 = ‘habitual drinkers with perceived control to refuse’. Subgroup 4 = ‘drinkers not in control’.

^*^ *P* < .05.

^**^ *P* < .01.

^***^ *P* < .001.

Furthermore, there was no significant interaction effect [*χ*^2^(3) = 1.35, *P* = .718], meaning that the change in excessive drinking volumes over time was similar between the four subgroups. Additional analyses showed significant interaction effects between subgroup, time, and gender [Wald *χ*^2^(8) = 35.09, *P* < .001] and between subgroup, time, and abstinence [Wald *χ*^2^(8) = 15.70, *P* < .05]. However, models incorporating these additional interaction effects performed worse in terms of goodness of fit measures than those without them. Therefore, we opted for models without these additional interaction effects.

## Discussion

### Key findings

This study examined the predictive value of subgroups for abstinence during a TAC and changes in drinking behaviour after a TAC. TAC subgroups differed in abstinence and in changes in drinking frequency and glasses per drinking day on weekdays. More specifically, ‘drinkers in control’ and ‘habitual drinkers with perceived control to refuse’ were more likely to abstain during the challenge than ‘ordinary drinkers’, whereas ‘drinkers not in control’ were less likely to abstain. Additionally, ‘drinkers in control’ showed smaller reductions in drinking frequency and glasses per drinking day on weekdays compared with ‘ordinary drinkers’. No significant differences between subgroups were found in changes in excessive drinking volumes and glasses per drinking day on weekends.

### Interpretation of key findings

Previous TAC research showed that abstinence was predicted by male gender, lower alcohol use, and higher drinking refusal self-efficacy (de Visser et al. 2016, de Visser and Nicholls 2020, de Visser and Piper 2020). This study, controlling for gender and drinking behaviour, found that TAC subgroups predicted abstinence during the TAC. Subgroup differences in drinking-refusal self-efficacy seemed to overlap considerably with the observed abstinence. Furthermore, ‘habitual drinkers with perceived control to refuse’ had higher rates of abstinence, possibly due to previous TAC participation improving their abstinence strategies (Kools et al. 2024). In contrast, ‘drinkers not in control’ struggled to stay abstinent, even when accounting for initial alcohol use, which aligns with prior findings on low drinking refusal self-efficacy (de Visser et al. 2016, de Visser and Nicholls 2020, de Visser and Piper 2020).

Consistent with previous research, all subgroups showed reductions in both drinking frequency, glasses per drinking day, and excessive drinking volume (de Visser et al. 2016). Yet, subgroups could only predict changes in drinking frequency and glasses per drinking day on weekdays, with only ‘drinkers in control’ having significantly smaller reductions than ‘ordinary drinkers’. This seems counterintuitive, due to the more ‘beneficial’ subgroup configurations among ‘drinkers in control’, including higher drinking refusal self-efficacy and lower craving and behavioural automaticity (Kools et al. 2024). These configurations would typically predict lower alcohol use and/or lower rates of relapse to drinking (Webb and Sheeran 2006, Schneekloth et al. 2012, de Visser et al. 2016, Gardner et al. 2020, de Ternay et al. 2022). One explanation for this difference between these two subgroups might be the relative larger initial ‘room for improvement’ for ‘ordinary drinkers’ than ‘drinkers in control’ (i.e. 77.9% versus 3.7% of participants drinking four or more days per week and 15.7% versus 3.0% of participants drinking five or more glasses per drinking day on weekdays).

There might be multiple other explanations for the observed subgroup differences in both abstinence and changes in drinking behaviour, including factors that have not been measured in the present study. For example, previous research suggests that factors such as changes in drinking motives, social influences, refusal self-efficacy, habits, alcohol-related identity, and awareness of drinking patterns may contribute to changes in drinking behaviour after TACs (de Visser et al. 2016, Butters et al. 2023, Thienpondt et al. 2025).

Another potential influence on changes in drinking behaviour is differences in motivation for long-term behaviour change. While participants in TACs do not necessarily need to have such motivation, subgroups with lower alcohol use might simply have lower motivation to change. Indeed, previous research suggested that both self-efficacy and motivation to change are necessary for long-term changes in drinking behaviour (Prochaska et al. 1992, Kuerbis et al. 2013, Müller et al. 2019). Furthermore, motivation to change was only related to reductions in alcohol use among drinkers with (very) high alcohol use (de Vocht et al. 2018). Therefore, the difference could be explained by a potential higher motivation to change drinking behaviour among ‘ordinary drinkers’ combined with their significantly higher alcohol use compared with ‘drinkers in control’ (Kools et al. 2024). Future research should explore the relative contributions of these factors as potential mechanisms of change.

This study aimed to assess the potential value of TACs in promoting changes in drinking behaviour for public health purposes. In that context, the study’s findings may suggest that the participation value for ‘drinkers not in control’ and ‘drinkers in control’ in its current form might be limited. First, despite reductions in alcohol use among ‘drinkers not in control’, this subgroup still showed higher alcohol use postchallenge (e.g. 8.7% drinks four or more days per week and 58.7% drinks excessive volumes at follow-up). This, combined with their struggles with abstinence, suggests that the current form of TAC may not fully leverage their potential for improvement, limiting its public health impact.

Second, ‘drinkers in control’ already had low alcohol use and minimal room for further reduction. Their participation in TACs may not offer significant benefits in terms of changing drinking behaviour. The participation value for the other two subgroups (i.e. ‘ordinary drinkers’ and ‘habitual drinkers with perceived control to refuse’) seemed more profound. Future research could explore whether changes in drinking behaviour for these subgroups (and particularly for ‘drinkers not in control’) can be optimized by providing personalized support. Since tailored health behaviour change interventions are more effective than one-size-fits-all approaches (Norman et al. 2007, Neville et al. 2009, Boumans et al. 2022), implementing tailored preventive support earlier could reduce abstinence lapses and improve intervention effectiveness. Strategies could address subgroup-specific barriers, such as managing social pressure and teaching emotional regulation techniques or craving-reducing habits.

### Strengths and limitations

A strength of this study was that, for the first time, research has been conducted into subgroups of TAC participants with its predictive value to intervention success (i.e. abstinence and changes in drinking behaviour), using a relatively large sample size. There were, however, also some limitations. First, these results should be interpreted cautiously within the broader context of TAC participants and the general population. Differences between this sample and registered participants who did not complete the questionnaire are unknown, as we are unaware of potential response bias. Research on response bias in voluntary recruitment shows that individuals who are female, older, highly educated, and consume less alcohol are more likely to participate (Cheung et al. 2017). Therefore, the prevalence of surveyed characteristics might be skewed in comparison to the full group of TAC participants. Furthermore, it is important to interpret the findings in the context of TAC participants, who are likely not representative of the overall drinking population, as suggested by other studies (de Visser and Piper 2020, Lespine et al. 2024).

Second, there was an attrition rate of 47.7% from baseline measurement to 8-month follow-up, with various differences between participants who did and did not complete the follow-up questionnaire (e.g. participants completing follow-up were older, reported less excessive drinking volumes at baseline, and were more likely to be retired, have a high educational level and be ‘habitual drinkers with perceived control to refuse’). This indicates selective attrition, which may limit the generalizability of our findings. It is possible that those who did not stay abstinent during the challenge and/or did not change drinking behaviour felt less inclined to participate in follow-up research, potentially leading to a more optimistic representation of the TAC. Methodologically, we chose not to adjust for attrition, as this could have introduced—rather than reduced—bias due to the selective nature of attrition.

## Conclusions

This study demonstrated the predictive value of subgroups for abstinence but found limited predictive value for changes in drinking behaviour after the challenge. The participation value for ‘drinkers not in control’ and ‘drinkers in control’ might be questionable, due to limited longer-term changes in drinking behaviour (i.e. due to remaining higher alcohol use and lack of room for improvement, respectively). Future research could explore whether changes in drinking behaviour can be optimized by providing personalized support.

## Data Availability

Data underlying this article are available on reasonable request to the corresponding author.
